# Current status of genetic urinary biomarkers for surveillance of non-muscle invasive bladder cancer: a systematic review

**DOI:** 10.1186/s12894-020-00670-x

**Published:** 2020-07-14

**Authors:** F. Lozano, C. X. Raventos, A. Carrion, E. Trilla, J. Morote

**Affiliations:** 1 Urology Department, Vall d’Hebron University Hospital, Pg. Vall d’Hebron 119-129, 08035 Barcelona, Spain; 2 Universitat Autònoma de Barcelona, Barcelona, Spain

**Keywords:** Biomarkers, Surveillance, Bladder, Genetic

## Abstract

**Background:**

Genetic biomarkers are a promising and growing field in the management of bladder cancer in all stages. The aim of this paper is to understand the role of genetic urinary biomarkers in the follow up of patients with non muscle invasive bladder cancer where there is increasing evidence that they can play a role in avoiding invasive techniques.

**Methods:**

Following PRISMA criteria, we have performed a systematic review. The search yielded 164 unique articles, of which 21 articles were included involving a total of 7261 patients. Sixteen of the articles were DNA based biomarkers, analyzing different methylations, microsatellite aberrations and gene mutations. Five articles studied the role of RNA based biomarkers, based on measuring levels of different combinations of mRNA. QUADAS2 critical evaluation of each paper has been reported.

**Results:**

There are not randomized control trials comparing any biomarker with the gold standard follow-up, and the level of evidence is 2B in almost all the studies. Negative predictive value varies between 55 and 98.5%, being superior in RNA based biomarkers.

**Conclusions:**

Although cystoscopy and cytology are the gold standard for non muscle invasive bladder cancer surveillance, genetic urinary biomarkers are a promising tool to avoid invasive explorations to the patients with a safe profile of similar sensitivity and negative predictive value. The accuracy that genetic biomarkers can offer should be taken into account to modify the paradigm of surveillance in non muscle invasive bladder cancer patients, especially in high-risk ones where many invasive explorations are recommended and biomarkers experiment better results.

## Background

Bladder cancer (BC) is the fifth most common neoplasm worldwide, with more than 54,000 new cases estimated per year in the United States alone [1] . BC is a heterogeneous tumour that is associated with very high economic costs and a substantial impact on patients’ quality of life owing to its characteristically high risk of recurrence and the complexity of follow up [2]. Guidelines from the European Association of Urology (EAU) and the American Urological Association (AUA) suggest a combination of cystoscopy, cytology and imaging for the surveillance of patients with non-muscle invasive bladder cancer (NMIBC) [3, 4]. Cystoscopy is an invasive procedure that carries the risks of painful micturition, urinary frequency and macroscopic haematuria of 50, 37 and 19%, respectively [5], while cytology has a very low sensitivity, especially for low-grade tumours [6, 7] .

For this reason there has been an increase in research over the past years into urinary biomarkers for the three scenarios of haematuria, diagnosis and surveillance. The role of these new tests is to increase the sensitivity and the specificity of the available gold-standard techniques, while sparing the patient the discomfort of an invasive test and its potential complications. Although many types of urinary biomarkers have been investigated, biomarkers that use genetic materials such as DNA and RNA seem to be the most promising due to their potential to identify a genetic signature. Such a signature would not only prove useful in disease detection and follow-up but also in the facilitation of more precise treatment by avoiding unhelpful therapies that may delay the best oncological pathway.

The field of urinary biomarker research in BC is focused on balancing a non-invasive, safe method with a cost-effective strategy that can be used to improve the sensitivity of bladder tumour detection in the initial phase of the disease and during patient follow-up, compared with the current gold standard.

Selecting a biomarker must be based on the given scenario and follow the principles of the international guidelines [8, 9] . The current literature clearly differentiates between different biomarker tests and characteristics depending on whether the BC is low or high risk. For low-risk tumours, marker-guided testing of lesions is suggested to detect possible progression to high-risk tumours. For high-risk tumours, however, where early detection is the main objective, selection of high-sensitivity biomarkers is recommended [9].

The aim of this study is to analyse the current literature for the use of genetic urinary biomarkers in the surveillance of NMIBC.

## Methods

FLP performed a bibliographic search of Medline (http://www.ncbi.nlm.nih.gov), Embase (http://www.embase.com) and the Cochrane library (http://www.cochrane.org) up to March 2020. MeSH terms used were Bladder cancer AND surveillance AND biomarkers AND DNA OR RNA OR methylation, yielding 2241 articles. After that, two authors (FLP and CXR) screened all published original articles appearing in the above search for eligibility. Studies using genetic urinary biomarkers for surveillance in non muscle invasive bladder cancer in humans were selected. Studies were excluded if they were not original research papers, used a language other than English, had less than 20 patients or did not report biomarker performance in terms of sensitivity, specificity, or area under the curve (AUC); or reported the performance of genetic markers only in combination with other factors (clinical data or non genetic biomarkers), yielding 164 articles.

**Fig. 1:**
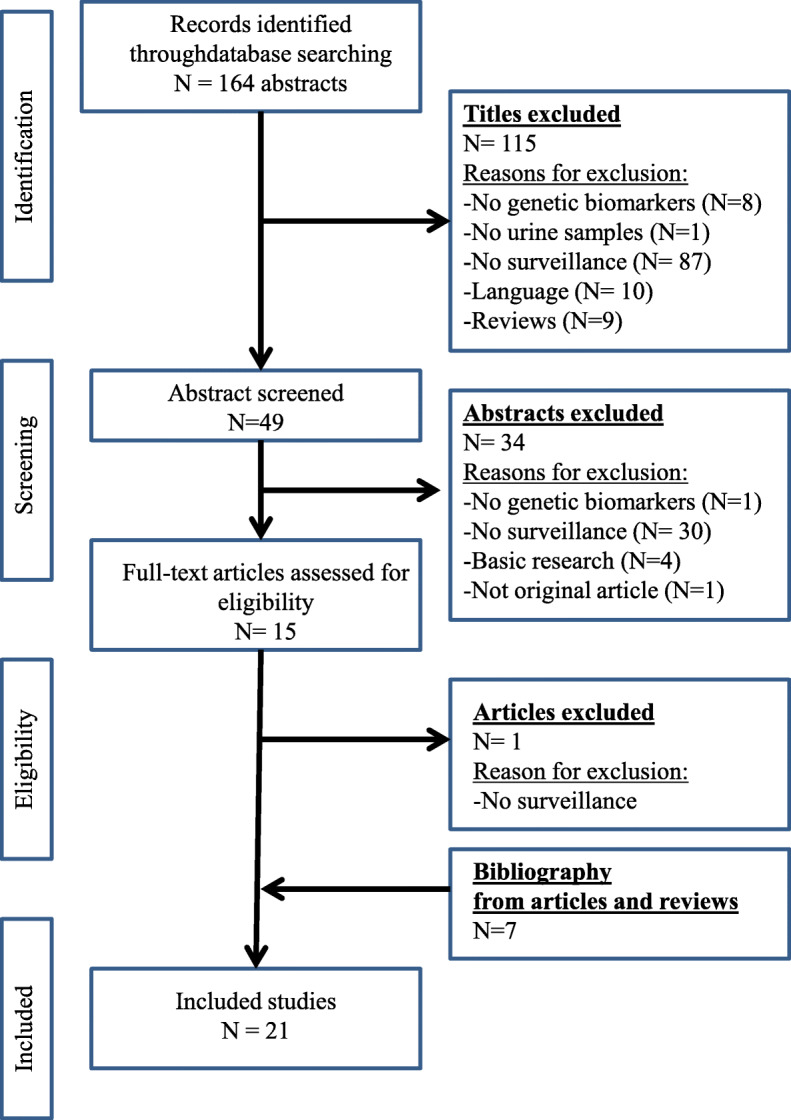
Flow chart following PRISMA criteria

After applying the PRISMA (Preferred Reporting Items for Systematic Reviews and Meta-analyses) criteria narrowed this down to 21 original articles (Fig. 1).

**Fig. 2:**
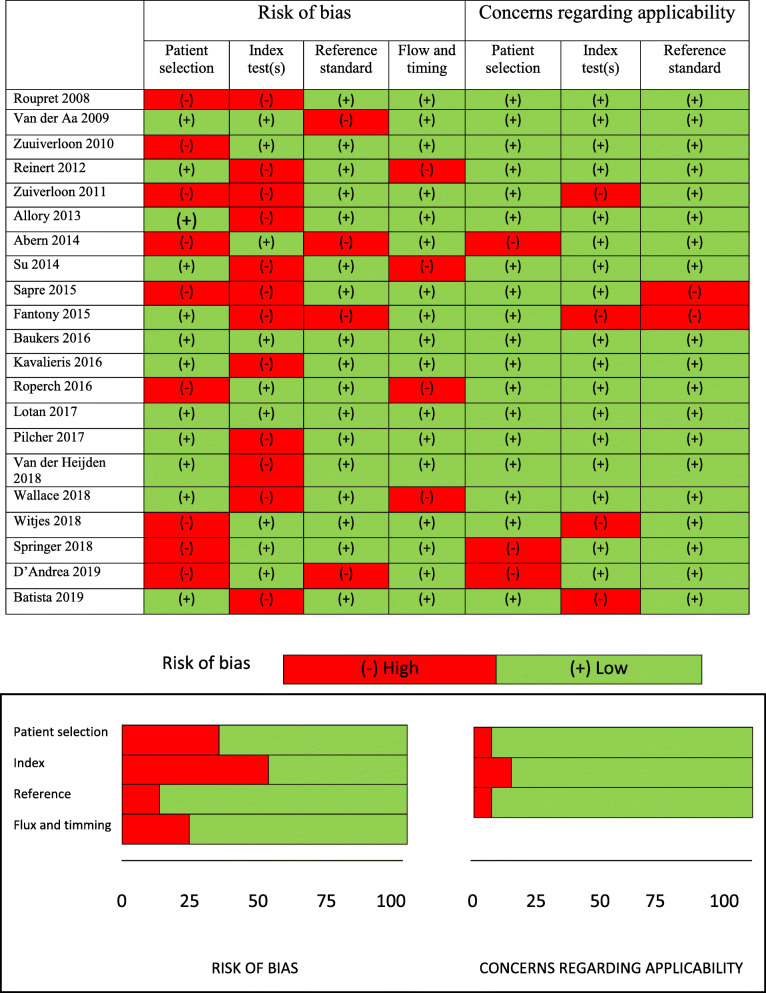
QUADAS 2 table evaluating quality of the studies

After reviewing each of the selected articles using the REMARK checklist [10], we designed a QUADAS-2 table to evaluate each study’s risk of bias and quality (Fig. 2). The main bias observed was reference to the index test. Although we identified a 50% risk of bias, most of the articles reviewed met most of the QUADAS-2 criteria [11], using the four considerations (patient selection, index, reference and flux and timming) which suggested that the studies were of moderately high quality.

**Fig. 3:**
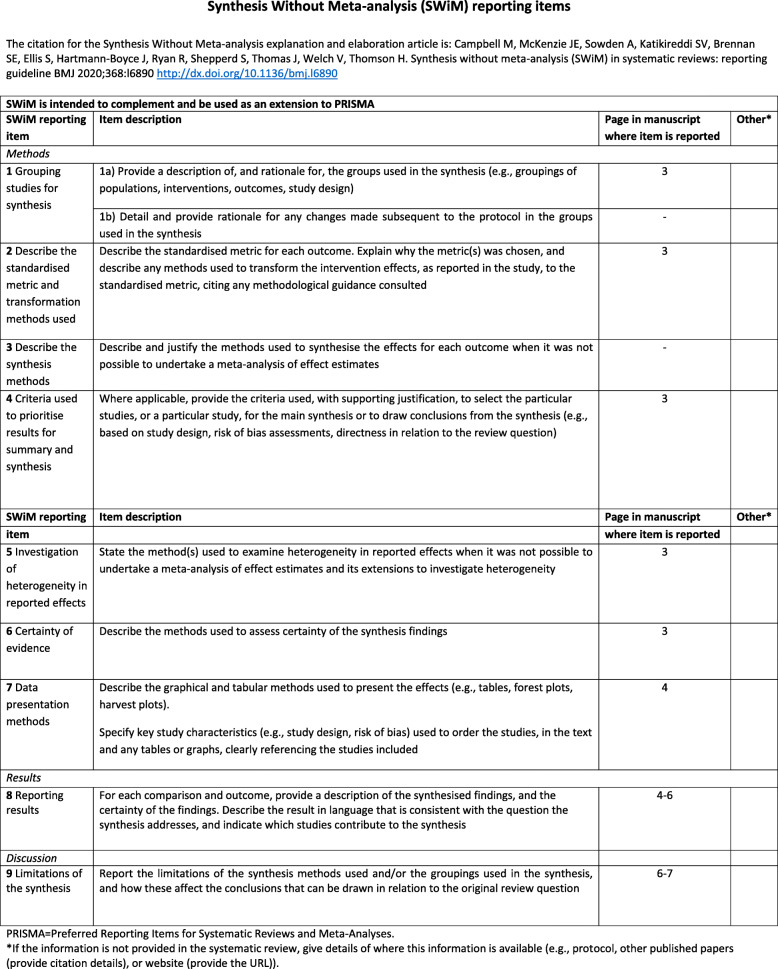
Synthesis Without Meta-analysis (SWiM) reporting items

We have also used the SWIM (Synthesis without meta-analysis) reporting guideline [12] to clarify the reporting methodology of the article (Fig. 3). The evidence was reported in terms of sensitivity and specificity for each biomarker (for low grade and high grade tumors in case of studies that indicated that subestratification). Area under de curve has been informed in eleven of the seventeen studies. We have also reported negative and positive predictive value of the markers. Recurrence rate has been calculated using the positive cases (positive pathology) and the total number of samples. We have prioritized articles identified as low risk of bias based on QUADAS-2 table to draw the conclusions of this review. We have also performed exploratory analyses to determine whether different study characteristics varied the effects of the interventions. Almost all the studies accepted as confirmed positive case if there is a pathology report. Some studies generate artificial cohort. We examined whether this different type of targeted behaviour modified, on average, the effect of the interventions.

## Results

In order to obtain a practical and visual description of the different studies, we divided the biomarkers by method into DNA-based (Table 1) and RNA-based (Table 2) tests.

**Table 1:** DNA based biomarkers used in follow-up for non muscle invasive bladder cancer patients

Reference	Patients/samples	Recurrence rate	Sensitivity(%)	Specificity(%)	NPV (%)	PPV (%)	AUC	Method	Markers
Roupret *et al* 2008 [13]	40/40	38%	80 (microsatellite) 86 (methylation) 85 (combination)	68 (microsat) 8 (methyl) 86 (combination)			0.81 (microsat) 0.44 (methyl)	DNA PCR	Microsatellite^a^ vs methylation^b^
Van der Aa *et al* 2009 [14]	228/815	10.3%	58	73	94	61-77	NA	DNA PCR	Microsatellite + FGFR3 mutation
Zuiverloon *et al* 2010 [15]	134/463	9.7%	58	NA	89	25	NA	DNA PCR	FGFR3 mutations^c^
Reinert *et al* 2012 [16]	158/206	67.4%	87-94	28-47	55-78	72-78	0.68-0.78	DNA PCR	Methylation ^d^
Zuiverloon *et al* 2012 [17]	NA/94	69.1%	72.3	55.2	NA	NA	NA	DNA PCR	Methylation genes APC_a , TERT_a , TER _b ,EDNRB
Allory *et al* 2013 [18]	194/395	44.8%	19(FGFR3) 42(TERT) 50(FGFR3+TERT)	73 (TERT) 90(FGFR3) 71(FGFR3+TERT)	NA	NA	NA	DNA PCR	Gene mutations (TERT and FGFR3)
Abern *et al* 2014 [19]	111/111	21.6%	75-79	63-71	92	37-42	0.74 (TWIST1) 0.68 (NID2)	DNA PCR	Methylation genes TWIST1, NID2
Su *et al* 2014 [20]	90/368	37.7%	80	97	NA	NA	0.95	DNA PCR	Hyper and hypomethylated genes (SOX1, IRAK3, L1-MET)
Fantony *et al* 2015 [21]	126/126	25%	58-67	61-69	83-85	36-38	0.66 (TWIST1) 0.63 (NID2)	DNA PCR	Methylation genes TWIST1, NID2
Beukers *et al* 2016 [22]	NA/2191	64%	57 (LG) 72 (HG)	59% LG	NA	NA	NA	DNA PCR	FGFR3 mutation, TERT mutation and OTX1 methylation
Roperch *et al* 2016 [23]	158/613	45.5%	94.5 96 (HG)	75.9	98.5	NA	0.82	DNA PCR	FGFR3 mutation +DNA methylation HS3ST2, SLIT2 and SEPTIN9
Van der Heijden *et al* 2018 [24]	NA/458	37.7%	90	31	82	50	0.74	DNA PCR	DNA gene Methylation (CFTR, SALL3, TWIST1)
Witjes *et al* 2018 [25]	353/353	13%	68.2 92.6 (HG)	88	95.1 99.3( HG)	44.8	0.82	DNA PCR	15 DNA methylation genes (Epicheck®)
Springer *et al* 2018 [26]	322/322	58%	68 71 (HG)	80	NA	NA	NA	DNA PCR	10 gen mutations^e^ plus detection of aneuploidy (UroSEEK®)
D’Andrea *et al* 2019 [27]	357/357	13.7%	67.3 88.9 (HG)	88 88(HG)	94 99 (HG)	47 30 (HG)	85.9	DNA PCR	15 DNA methylation genes (Epicheck®)
Batista *et al* 2019 [28]	122/122	28%	73.5	73.2	NA	NA	NA	DNA PCR	TERT promoter and FGFR3 mutations (Uromonitor®)

*LG* low grade, *HG* high grade, *NA* not allowed

^a^FGA (4q28), D4S171(4q35)), 5 (ACTBP2(5q14)), 9 (D9S162 (9p), IFNA (9p21)), 14 (MJD52(14q32)), 16 (D16S310 (16q21)) and 18 (D18S51 (18q21), MBP (18qter).

^b^(RASSF1a (3p21.3),E-cadherin (16q22.1), APC (5q21), DAPK (9q22.1), MGMT (10q26), BCL2 (18q21.33), h-TERT (5p15.33), EDNRB (13q22), WIF-1 (12q14.3), TNFRSF25 (1p36.31), IGFBP3 (7p13))

^c^R248C and S249C (exon 7); G372C,S373C, Y375C, G382R, and A393E (exon 10); and K652M, K652T, K652E, and K652Q (exon 15)

^d^EOMES, HOXA9, POU4F2, TWIST1, VIM, ZNF154

^e^FGFR3, TP53, CDKN2A, ERBB2, HRAS, KRAS, PIK3CA, MET, VHL, MLL and TERT promoter.

**Table 2:** RNA based biomarkers used in follow-up for non muscle invasive bladder cancer patients

Reference	Patients/samples	Recurrence rate	Sensitivity	Specificity	NPV	PPV	AUC	Method	Markers
Sapre *et al* 2016 [29]	131/131	NA	88	48	75	63	0.74	miRNA PCR	6 miRNA signature^a^
Kavalieris *et al* 2017 [30]	736/1036	15.1%	92	NA	96	NA	0.73	mRNA PCR	5 genes mRNA expression (Cx Bladder Monitor® )^b^
Lotan *et al* 2017 [31]	748/1016	14.8%	91 95 (HG)	NA	96	NA	NA	mRNA PCR	5 genes mRNA expression (Cx Bladder Monitor® )^b^ vs NMP22 ELISA vs NMP22 BladderChek
Pilcher *et al* 2018 [32]	140/155	30.7%	84 100 (HG)	91	93	72	0.87	mRNA RT-PCR	ABL1, CRH, IGF2, UPK1B, ANXA10 (Xpert Bladder Cancer Monitor®)
Wallace *et al* 2018 [33]	370/370	13.2%	73 83 (HG)	77	92	44	0.87	mRNA RT-qPCR	ABL1, CRH, IGF2, ANXA10, UPK1B (Xpert Bladder Cancer Monitor®)

*HG* high grade, *NA* not allowed

^a^miR16, miR200c, miR205, miR21, miR221 and miR34a

^b^IGFBP5, HOXA13, MDK, CDK1, CXCR2

### DNA tests (Table 1)

DNA tests used for surveillance are based on microsatellite analysis (MA). They are employed to detect loss of heterozygosity, gene methylation levels and gene mutations in cells collected from urine.

Microsatellite markers are highly polymorphic tandem repeat DNA sequences distributed throughout the genome and easily amplifiable by standard polymerase chain reaction [34] . Rouprêt et al. [13] compared this biomarker with methylation biomarkers in a comparative cohort study of 40 patients. In this study, MA appeared to yield better results for detecting recurrences (AUC 0.81 vs 0.44). When a Bayesian network analysis was performed that combined variables and biomarkers, the panel of markers generated a sensitivity of 85% and a specificity of 86%. Van der Aa et al. [14] designed a multicentre study to evaluate the clinical utility of MA in low-grade tumours in combination with *FGFR3* mutations described previously [35]. The sensitivity in this study was 58% and the specificity 73%, with a negative predictive value of 94%.

DNA methylation has been recognized to be important in the developmental biology and cancer aetiology of many neoplasms [36–38]. DNA methylation is an epigenetic marker that mainly affects CpG dinucleotides. These dinucleotides are distributed throughout the genome and usually have a normal methylation status. Hypermethylation of CpG dinucleotides in the promoter regions of tumour suppressor genes can repress their transcription in human cells [39, 40]. Methylation status is one of the most studied biomarkers in the follow-up scenario because it is both chemically stable and quantifiable [41]. Zuiverloon et al. [17] developed a retrospective four-step test, selecting methylation of the *APC_a*, *TERT_a*, *TER_b* and *EDNRB* genes as the combination providing a higher sensitivity and specificity (63.3 and 58.3%, respectively) than other combinations investigated in this study. Based on their previous study [42], Reinert et al. evaluated the methylation of *EOMES*, *HOXA9*, *POU4F2*, *TWIST1*, *VIM* and *ZNF154*. Their study consisted of a first step, validating the markers and establishing the cut-off levels, and a second step in the surveillance scenario excluding those patients who showed no aberrant methylation of their tumour marker genes. The authors reported a sensitivity of between 87 and 94% and a specificity ranging from 43 to 67%. Combining the different biomarkers did not improve the accuracy of the test [16]. Su et al. [20] tested six DNA methylation markers before building a model with *SOX1*, *IRAK3* and *L1-MET* as the best combination to detect recurrences. Using this model they obtaining a sensitivity of 80% and a very high specificity of 97%. Roperch et al. [23] combined four different *FGFR3* mutations and eighteen methylation markers based on the literature [43, 44]. Finally, they selected three of these markers (the genes *HS3ST2*, *SLIT2* and *SEPTIN9*) for combination with the *FGFR3* mutations in a logistic regression model, obtaining a sensitivity of 94.5% (96% in high-grade tumours) and a specificity of 75.9%. Van der Heijden et al. [24] evaluated seven selected genes that are found at significantly increased levels in the urine sediment from patients with BC. After testing a training set, they selected the *CFTR*, *SALL3* and *TWIST1* genes for validation in a large series (458 samples) and obtained a sensitivity of 90% (96% in combination with cytology). Witjes et al. [25] evaluated a combination of 15 methylated genes (Epicheck®), obtaining a sensitivity of 68.2% (92.6% for high-grade tumours) and a specificity of 88%. D’Andrea et al. [27] published another multicentric and independent study using the same test, supporting the sensitivity (67.3, 88.9% for high grade) and specificity (88%) described in the previous publication by Witjes. Abern [19] studied the role of two methylated genes, TWIST1 and NID2 based on Renard work [45] due to their high sensitivity and specificity for urothelial carcinoma. They observed that TWIST1 methylation had better AUA than NID2 or the combination of both genes. They also showed that adjusting the thresholds, the test had a sensitivity and specificity of 75 and 71%, respectively. Fantony et al. [21] published a more recent multi-institutional study using the same methylated genes, obtaining similar conclusions and results of sensitivity (58–67%) and specificity (61–69%). In this paper, prior BCG treatment for NMIBC reduced the accuracy of the test.

Many of the gene mutations investigated are related to the carcinogenesis of urothelial carcinomas, which are among the most heterogeneous tumours [46]. One of the most studied among these genes is fibroblast growth factor receptor 3 (*FGFR3*), mutations of which are found in almost 80% of the low-grade tumours and associated with a good prognosis [35, 47].

Zuiverloon et al. [15] evaluated this marker in non-high grade tumours, achieving a sensitivity of 58%. Beukers et al. [22] combined *FGFR3* mutation with *TERT* mutation and *OTX1* gene methylation in a large prospective European cohort study, obtaining a sensitivity of 57% for low-grade and 72% for high-grade BC. Allory investigated the role of telomerase reverse transcriptase (TERT) promoter mutations, frequently founded in many other non urothelial tumors [48] in combination with FGFR3 mutation [18]. This study showed that combination of TERT and FGFR3 has higher sensitivity (50%) than TERT or FGFR3 individually. Moreover, FGFR3 had higher specificity than TERT mutation.

In a more recent multicentric study, Batista et al. [48] have developed a biomarker based on two TERT mutations (c. 1-124C > T and c.1-146C > T) plus FGFR3 (p.R248C and p.S249C) hotspot mutations. After a technical validation of the test, they achieved a73.5% of sensitivity and 93.2% of specificity. Springer et al. [26] have also analyzed mutations in TERT promoter, mutations in FGFR3 in combination with other nine gen mutations (TP53, CDKN2A, ERBB2, HRAS, KRAS, PIK3CA, MET, VHL, MLL) plus detection of aneuploidy,an abnormal chromosome number, that has been estimated to be present in > 90% of the cancer of most histopathologic types [49]. They found that this combination could detect recurrences with a sensitivity of 68% and a specificity of 80%.

### RNA tests (Table 2)

RNA biomarkers are less well studied in the field of BC surveillance.

MicroRNAs (miRNAs) are B22-nucleotide long, single-stranded, non-coding RNAs that bind to complementary ‘seed’ regions found in the 30-untranslated region of particular messenger RNA (mRNA) species. MiRNAs can modulate the expression of their mRNA targets and are involved in many physiological processes, but also in carcinogenesis [50]. Sapre et al. [29] evaluated a 12-miRNA-panel test, with the aim of selecting the minimum number of miRNAs necessary to achieve an accurate prediction. They found that a selection of six miRNAs (miR16, miR200c, miR205, miR21, miR221 and miR34a) provided a sensitivity of 88% and a specificity of 48%.

Kavalieris et al. [30] and Lotan et al. [31] tested a combination of five mRNAs (*IGFBP5*, *HOXA13*, *MDK*, *CDK1*, *CXCR2*), commercially available under the brand name Cx Bladder Monitor®, and reported highly consistent results for the evaluation of the mRNA expression from the five genes. The studies included a scoring system, based on variables such as previous tumour status (primary or recurrent) and time since previous tumour in years, to classify the test as positive or negative. The authors reported sensitivities between 91 and 92% (95% in high-grade tumours) and a negative predictive value of 96%.

Wallace et al. [33] and Pichler et al. [32] tested the Xpert BC Monitor®, a commercial kit that measures five target mRNAs (*ABL1*, *CRH*, *IGF2*, *UPK1B*, *ANXA10*), in a population of 510 patients and obtained sensitivities between 73 and 84% (100% in high-grade tumours) and a negative predictive value of 92–93%. They also confirmed that cytology did not enhance diagnostic accuracy.

## Discussion

Biomarker investigation is a growing field in the management of NMIBC. Many of the investigations are used in different scenarios: diagnosis, surveillance, and risk stratification of patients with NMIBC. Although many molecular marker tests have been developed to improve diagnostic and surveillance accuracy, with some having been approved by the US Food and Drug Administration, none of the currently available tests have been accepted or incorporated into the follow-up algorithms described in the guidelines [51].

Biomarkers can be divided into cellular, protein and genetic markers. The latter are the most recent and, in contrast to cytology, have the advantages of being reliable, easy to perform, and objective.

In fact, they perform significantly better in BC because thousands of genetic changes can be detected accurately and simultaneously compared with the lower-throughput protein-based biomarkers. As aberrant DNA methylation also occurs in non-malignant tissue it is not pathognomonic of malignancy and genetic methylation cannot be used to distinguish between cancer cells and other pre- or non-neoplastic cells [52]. However, this genetic biomarker has the benefits of always occurring in the same DNA location and chemical stability which make it easier to detect than gene mutations.

Protein-based and cell-based biomarkers are also more likely to be affected by benign conditions such as infection, inflammation and bladder treatments.

To date the gold standard for these cases, as outlined in the guidelines, is to use cystoscopy and cytology. Cystoscopy is an invasive procedure that may be associated with pain and discomfort [5]. Moreover, cystoscopy does not detect all lesions and is subject to the experience of the urologist or nurse [53]. Voided urine cytology needs trained cytopathologists and has the potential for inter-observer variability.

Researchers who are developing urinary biomarkers are looking for high sensitivity and a high negative predictive value. This profile is of special interest in the follow-up scenario because the aim of these tests is to reduce the number of cystoscopies by alternating the procedures, rather than avoiding cystoscopy altogether. Thus, cystoscopy will only be performed when the urine test is positive (urine-first strategy).

One of the major limitations of the use of DNA- or mRNA-based techniques is the difficulty in obtaining sufficiently large quantities of high-quality RNA from voided urine. In terms of monitoring, another limitation of non-invasive urine biomarkers is their low sensitivity, particularly for early-stage and low-grade tumours that account for a significant proportion of recurrences.

Almost all the studies had a high percentage of ‘false’-positive urine tests for the detection of concomitant recurrences, resulting in low specificity. In many articles, the authors justified these percentages with the well-known phenomenon of the anticipatory effect, i.e. the urine test detects recurrent tumours earlier than cystoscopy. It is accepted in the literature that anticipatory detection would include recurrences that occur within the next 18 months after a positive biomarker test [54]. In any case, performing a cystoscopy because of a false positive is more acceptable than missing a tumour because of a false negative.

Other limitations of the studies included in this review are the retrospective nature of some of the cohorts used for the outcome analysis, artificial oversampling of the recurrence rate by recruiting patients scheduled for transurethral resection of a proven bladder tumour, and using the same population for the training and the validation sets, which increases the possibility that the performance of the biomarker may be artificially inflated due to over-fitting.

In this review, most of the biomarker tests are dichotomous, providing either positive (tumour detected) or negative (no tumour) test results. However, giving a numerical prediction of the probability of a recurrent tumour may be more helpful to urologists in terms of their decision-making.

Moreover, there is a lack of uniformity in the design of the studies. Some of the works describe surveillance programs but they create the cohorts. Many of the biomarkers tested need clinical information to complete an algorithm and yield a positive or negative result, which increases subjectivity and decreases the homogeneity of results.

The main limitations were the lack of randomized control trials and the diverse study outcomes, which made meta-analysis impossible to perform. Comparison between sensitivity and specificity of different biomarkers may generate a bias due to the different incidence and different cohort.

Literature lacks of direct comparison between urinary biomarkers and gold standard maybe due to commercial interests.

## Conclusion

BC is one of the most expensive tumours due to its high recurrence rate and the costs of the follow-up protocols.

This is the reason why there is an increased interest in biomarkers, in order to reduce the number of exploratory investigations and improve the quality of life of patients with BC. In this review, there are some genetic biomarkers with higher negative predictive value and sensitivity, especially for high-grade tumours, compared to the gold standard. European and US guidelines still recommend cystoscopy and cytology for follow-up. Genetic urinary biomarkers are a very heterogeneous group of test that nowadays cannot replace the standard pathway of surveillance with cystoscopy and cytology. Although there are some ongoing clinical trials comparing both options, there is no level 1 evidence to support their recommendation instead of the gold standard.

## Data Availability

All data generated or analysed during this study are included in this published article (and its supplementary information files).

## Abbreviations

PRISMA: Preferred reporting items for systematic reviews and meta-analyses
QUADAS: Quality Assessment of Diagnostic Accuracy Studies
BC: Bladder cancer
EAU: European Association of Urology
AUA: American Urological Association
NMIBC: Non-muscle invasive bladder cancer
MA: Microsatellite analysis
TERT: Telomerase reverse transcriptase
MiRNA: MicroRNAs
mRNA: Messenger RNA
